# Genome-wide characterization of ascorbate peroxidase gene family in pepper (*Capsicum annuum* L.) in response to multiple abiotic stresses

**DOI:** 10.3389/fpls.2023.1189020

**Published:** 2023-05-08

**Authors:** Xin Pang, Jun Chen, Yang Xu, Jia Liu, Yangmin Zhong, Linlin Wang, Jiaqiu Zheng, Hongjian Wan

**Affiliations:** ^1^ Suzhou Polytechnic Institute of Agriculture, Suzhou, China; ^2^ State Key Laboratory for Managing Biotic and Chemical Threats to the Quality and Safety of Agro-Products, Institute of Vegetables, Zhejiang Academy of Agricultural Sciences, Hangzhou, China; ^3^ Wulanchabu Academy of Agricultural and Husbandry Sciences, Wulanchabu, China; ^4^ Institute of Crops, Lishui Academy of Agricultural and Forestry Sciences, Lishui, China; ^5^ Jiangsu Coastal Area Institute of Agricultural Sciences, Yancheng, China

**Keywords:** pepper, ascorbate peroxidase, bioinformatics analysis, gene expression, phylogenetic relationship

## Abstract

Pepper is widely grown all over the world, so it faces many abiotic stresses, such as drought, high temperature, low temperature, salt damage, and so on. Stresses causing the accumulation of reactive oxidative species (ROS) in plants are removed by antioxidant defense systems, and ascorbate peroxidase (APX) is an important antioxidant enzyme. Therefore, the present study performed genome-wide identification of the *APX* gene family in pepper. We identified nine members of the *APX* gene family in the pepper genome according to the APX proteins’ conserved domain in *Arabidopsis thaliana*. The physicochemical property analysis showed that *CaAPX3* had the longest protein sequence and the largest molecular weight of all genes, while *CaAPX9* had the shortest protein sequence and the smallest MW. The gene structure analysis showed that *CaAPXs* were composed of seven to 10 introns. The *CaAPX* genes were divided into four groups. The *APX* genes of groups I and IV were localized in the peroxisomes and chloroplasts, respectively; the group II genes were localized in the chloroplasts and mitochondria; and the group III genes were located in the cytoplasm and extracell. The conservative motif analysis showed that all *APX* genes in the pepper had motif 2, motif 3, and motif 5. The *APX* gene family members were distributed on five chromosomes (Chr. 2, 4, 6, 8, and 9). The *cis*-acting element analysis showed that most *CaAPX* genes contain a variety of *cis*-elements related to plant hormones and abiotic stress. RNA-seq expression analysis showed that the expression patterns of nine *APXs* were different in vegetative and reproductive organs at different growth and development stages. In addition, the qRT-PCR analysis of the *CaAPX* genes revealed significant differential expression in response to high temperature, low temperature, and salinity stresses in leaf tissue. In conclusion, our study identified the *APX* gene family members in the pepper and predicted the functions of this gene family, which would provide resources for further functional characterization of *CaAPX* genes.

## Introduction

Plants will be subjected to various environmental stresses during their growth and development, comprising both abiotic stresses such as drought, freezing, high temperature, heavy metal, and salinity and biotic stresses such as viruses and fungi (Bhat et al., 2019; Raza et al., 2020). Environmental stress can directly or indirectly destroy the reactive oxygen species (ROS) balance in plants, causing a series of stress responses in crops. ROS has dual functions: on the one hand, an appropriate amount of ROS is necessary for organisms to participate in the regulation of signal networks as signaling molecules. On the other hand, excessive reactive oxygen species will cause certain damage to the cell membrane and organelles of crops, and when they exceed a certain limit, they will affect the growth and development of individual plants and even lead to their death, which must be removed as soon as possible (Davletova et al., 2005; Foyer and Shigeoka, 2011). Therefore, the balance between the production and removal of reactive oxygen species in cells is very important. Plants maintain the dynamic balance of reactive oxygen species through enzymatic or nonenzymatic mechanisms and form a complete antioxidant system in the process of evolution (Shigeoka et al., 2002; Maruta et al., 2016).

The clearance mechanism includes the enzymatic scavenging system, such as ascorbate peroxidase (APX), catalase (CAT), glutathione peroxidase (GPX), superoxide dismutase (SOD), and other antioxidant enzymes (Faize et al., 2011). APX is a very effective H_2_O_2_ scavenger and has the highest affinity for H_2_O_2_ among all H_2_O_2_ metabolic enzymes (Huang et al., 2017). Plant APX belongs to the type I heme peroxidase and copper oxidase families and can reduce H_2_O_2_ to O_2_ and H_2_O using ASA as an electron donor through the ascorbic acid (ASA)-glutathione (GSH) cycle (Asada, 1999).

Ascorbate peroxidase (APX) is a protease encoded by members of a multi-gene family. According to the subcellular localization of their proteins, they can be divided into four categories: cytoplasmic APX, peroxidase APX, chloroplast APX, and mitochondrial APX (Shigeru et al., 2002; Apel and Hirt, 2004). Eight members of the *AtAPX* gene family have been identified in *Arabidopsis thaliana*, including three in the cytoplasm (*APX1*, *APX2*, and *APX6*), three in the peroxidase (*APX3*, *APX4*, and *APX5*), and two in the chloroplast (*sAPX* both in the mitochondrial and chloroplast, *tAPX* in the thylakoid membrane) (Panchuk et al., 2002; Chew et al., 2003; Mittler et al., 2004; Narendra et al., 2006). The *APX* gene family of rice has eight genes, including two cytoplasmic isoforms (*OsAPx1* and *OsAPx2*), two peroxidase isoforms (*OsAPx3* and *OsAPx4*), two mitochondrial isoforms (*OsAPx5* and *OsAPx6*), and two in the chloroplast (*OsAPx7* and *OsAPx8*) (Teixeira et al., 2004; Teixeira et al., 2006; Hong et al., 2007; Ribeiro et al., 2017). Tomato has seven genes encoding APX isoforms: three cytoplasmic isoforms, two peroxidase isoforms, and two chloroplast isoforms (Najami et al., 2008). Recently, as more and more plant genomes have been sequenced, genome-wide identification and functional analysis of *APX* have been accomplished, such as upland cotton (Gossypium hirsutum Linn.) (Tao et al., 2018), sorghum (*Sorghum bicolor* L.) (Akbudak et al., 2018), maize (Qu et al., 2020), kiwifruit (*Actinidia chinensis*) (Liao et al., 2020), and so on.

The *APX* gene plays an important role in the growth and development of different plants and their response to environmental stress. The expression of *AtAPX1* was significantly upregulated in various biological and abiotic stress responses (Zimmermann et al., 2004). APX2 plays an important role in regulating heat stress response at different stages of plant development (Schramm et al., 2006; Frank et al., 2009). Overexpression of the *AtAPX3* gene in tobacco increased the protective effect of transgenic plants against oxidative stress (Wang et al., 1999). *APX6* modulates the ROS signal cross-talk with hormone signals to properly execute the germination program in *Arabidopsis*, and the apx6 mutant exhibited a high level of ROS and reduced germination (Chen et al., 2014). Loss of function in *OsAPX2* showed semi-dwarf seedlings, yellow–green leaves, leaf lesion mimics, and seed sterility (Zhang et al., 2013). Transgenic plants overexpressing the *SbpAPX* gene showed enhanced salt and drought stress tolerance compared to wild-type plants (Singh et al., 2014). Another study found that *BcAPX* genes were overexpressed in transgenic *Arabidopsis*, and the expression of APX and the APX activity in transgenic lines were higher than in nontransgenic (NT) plants under high temperatures (Chiang et al., 2015).

Pepper is an important solanaceous vegetable and is widely cultivated and eaten both as a vegetable and as a spice. However, pepper is particularly vulnerable to a number of biotic and abiotic stresses in production, such as pathogens, drought, and cold temperatures, which can easily cause a production drop. The *APX* gene, as an important class of antioxidant enzymes, has rarely been reported. In this study, *APX* gene family members were identified from the pepper genome database, and the physicochemical properties of *APX* gene members were analyzed by the bioinformatics method. The expression of *APX* gene members in different tissues was analyzed by downloading the RNA-seq data, and the gene expression under abiotic stress was analyzed by the qRT-PCR technique. This study provided the scientific basis for further exploring the molecular mechanism of *APX* gene family members in pepper under abiotic stress.

## Materials and methods

### Identification of the *APX* gene family in pepper

All of the pepper genome sequence data were downloaded from the Solanaceae Genomics Network (http://solgenomics.net/). The local database of the pepper genome sequences was constructed by the Bioedit7.0 software. In order to retrieve all the members of the APX family in pepper, two methods were employed, including the BLASTP algorithm and the Hidden Markov model (HMM). First, for BLASTP, we used 31 APX amino acid sequences from *Arabidopsis thaliana*, rice, tomato, and potato as an inquiry with the *e*-value set to 1*e* ^−10^. The amino acid sequences of 23 APXs were retrieved from the PLAZA 4.0 (https://bioinformatics.psb.ugent.be/plaza/versions/) and Phytozome (https://phytozome.jgi.doe.gov/pz/portal.html). We then used the HMM profile of the APX domain (Pfam: PF00141), which was from the Pfam database (http://pfam.xfam.org), as a query to detect this local pepper database. Finally, nine *CaAPX* genes were identified by merging the two methods in the pepper genome.

### Characterization of *APX* gene family in pepper

Physicochemical properties of APX proteins, such as protein length, molecular weight, and isoelectric point were checked using the ExPASy server (www.expasy.org). The subcellular localization of the CaAPX proteins was predicted using the online Euk-mPLoc 2.0 (http://www.csbio.sjtu.edu.cn/bioinf/euk-multi-2/) (Chou and Shen, 2010). For the analysis of the structural feature, the Gene Structure Display Server (GSDS) (http://gsds.cbi.pku.edu.cn) online tool was adopted by comparing the CDS and genomic sequences of each *APX* gene (Hu et al., 2014). Conserved motif analyses of the *CaAPX* genes were then performed using the MEME tool (http://meme-suite.org/tools/meme), identifying a maximum of 10 motifs with a motif width range of 6 to 50 (Bailey et al., 2006). The chromosomal location of the *CaAPX* genes was determined from the SGN database. The map diagram showing the location of *APX* genes on chromosomes was drawn by implementing MapDraw V2.1 software.

### Multiple sequence alignment and phylogenetic analysis

To determine the evolutionary relationship of the *APX* gene family, a phylogenetic tree was carried out among *A. thaliana*, *Oryza sativa*, *Solanum lycopersicum*, *Solanum tuberosum*, and *Capsicum annuum* protein sequences. The sequence alignment was constructed using Clustal X (version 1.8) and a neighbor-joining method with 1,000 bootstrap replicates in MEGA 7.0 software (Kumar et al., 2016). Any gaps or missing data in the sequence treatment were applied to partial deletion, and then branch lengths were assigned *via* the pairwise calculations of genetic distances.

### *Cis*-element analysis in the *CaAPX* gene promoters

The 1-kb sequence upstream of the start code from each of the *CaAPX* genes was obtained from the SGN database. The *cis*-elements in the promoter regions of the individual gene were examined using the means of the PlantCARE server (http://bioinformatics.psb.ugent.be/webtools/plantcare/html/) (Lescot et al., 2002). The cis-element analysis figure was made by TBtools (Chen et al., 2020).

### Expression analysis of *CaAPX* genes in diverse tissues

In this study, RNA-seq data from the NCBI database (http://pepperhub.hzau.edu.cn/) were used to investigate expression patterns of putative *CaAPX* genes in different tissues of cultivated pepper (Zunla-1). Different tissues in cultivated pepper included root, stem, leaf, bud, flower, and F-Dev1-9. The heatmap was formed using Multi Experiment Viewer (MeV) software (Howe et al., 2010).

### Plant materials and stress treatments

This study’s samples used for stress treatment were collected from a typical cultivated variety, D50. The pepper plants were cultivated in a chamber under 14-h light (28°C to 30°C) and 10-h dark (18°C to 20°C) period conditions.

Seedlings at the six true leaf stages were used for all experiments. For cold treatment, seedlings were subjected to 4°C for 1, 3, 6, and 12 h. For heat shock treatment, seedlings were subjected to 42°C for 0.5, 1, 3, and 6 h. Plants were subjected to 26°C for control. For salt stress treatment, seedlings were subjected to 300 mM NaCl. Plants were subjected to sterile water for control. Three biological replicates for each treatment were carried out in the experiments. All of the samples were immediately frozen in liquid nitrogen and stored at −80°C for further experimentation.

### RNA isolation and quantitative real-time PCR analysis

The transcription profiles of *APX* genes in pepper were performed through qRT-PCR analysis. All primers used in the experiment were designed by the Primer 5.0 software and listed in **Supplementary Table S1**.

The total RNA isolation and cDNA synthesis was performed using Trizol (Tiangen, Beijing, China) and DNase I (Transgen, Beijing, China) according to manufacturer instructions. In total, 1 μg of each of the treated RNA samples was reverse transcribed using PrimeScriptTM RT reagent Kit for qPCR (Transgen, Beijing, China). A 20-μl qRT-PCR reaction mixture, which contained 10 μl of SYBR Green I, 1 μl of forward primer, 1 μl of reverse primer, 1 μl of diluted cDNA, and 7 μl of sterile distilled H_2_O, was prepared. The mixture was subjected to the following program: 35 cycles of 3 min at 95°C, 30 s at 58°C, and 20 s at 72°C (ABI Real-Time PCR System, USA). GAPDH was used as an internal control (Cheng et al., 2017), and the fold change in gene expression was calculated using the 2^−ΔΔCT^ method. Each qRT-PCR reaction was carried out using three biological and technical replicates.

## Results

### Identification of the *APX* gene family in the pepper genome

In the present study, a total of nine *APX* genes were recognized and designated as *CaAPX1* to *CaAPX9*, respectively, based on BLASTP examinations against the pepper genome employing 31 APX proteins (eight AtAPX proteins, eight OsAPX proteins, seven SlAPX proteins, and eight StAPXs) as inquiries (**Table 1**). The comprehensive information of the nine *CaAPX* genes (e.g., molecular weights (MW), isoelectric points (p*I*), numbers of exons/introns, and subcellular localization) is shown in **Table 1**. CaAPX amino acid residue lengths ranged from 245 aa (CaAPX9) to 353 aa (CaAPX3), MW ranged from 27.0 (CaAPX9) to 38.8 kDa (CaAPX3), and p*I* ranged from 5.43 (CaAPX6) to 9.49 (CaAPX4). The number of exons ranged from eight to 11, with two genes comprising eight exons, three genes comprising nine exons, two genes comprising 10 exons, and two genes consisting of 11 exons (**Table 1**). The subcellular localization results predicted that five APX (CaAPX1, CaAPX5, CaAPX6, CaAPX8, and CaAPX9) proteins are located in the peroxisome, three proteins (CaAPX2, CaAPX3, and CaAPX4) are located in chloroplast, two proteins (CaAPX3 and CaAPX4) are also located in the mitochondrion, and only one protein (CaAPX7) is located in the cytoplasm and extracell (**Table 1**).

**Table 1:** General information about the nine *CaAPX* genes.

Gene	Gene accession No.	Chromosome location	Length of protein (AA)	Predicted size (kDa)	Isoelectric point (p*I*)	No. of exons/introns	Subcellular localization
***CaAPX1***	**Capana02g002557**	**ch02:147290511.147293908**	**310**	**34.3**	**6.72**	**10/9**	**Peroxisome**
***CaAPX2***	**Capana04g000971**	**ch04:23506178.23513063**	**346**	**37.7**	**8.27**	**11/10**	**Chloroplast**
***CaAPX3***	**Capana04g002111**	**ch04:170729674.170735490**	**353**	**38.8**	**7.12**	**11/10**	**Chloroplast, mitochondrion**
***CaAPX4***	**Capana06g001732**	**ch06:48644312.48651554**	**304**	**32.9**	**9.49**	**8/7**	**Chloroplast, mitochondrion**
***CaAPX5***	**Capana06g002525**	**ch06:171804392.171807190**	**250**	**27.4**	**5.93**	**9/8**	**Peroxisome**
***CaAPX6***	**Capana06g002561**	**ch06:176301253.176303953**	**250**	**27.5**	**5.43**	**9/8**	**Peroxisome**
***CaAPX7***	**Capana08g000304**	**ch08:34207895.34214385**	**310**	**35.5**	**8.13**	**10/9**	**Cytoplasm, extracell**
***CaAPX8***	**Capana08g002729**	**ch08:151484880.151492967**	**287**	**31.6**	**7.10**	**9/8**	**Peroxisome**
***CaAPX9***	**Capana09g001881**	**ch09:213803706.213807771**	**245**	**27.0**	**6.00**	**8/7**	**Peroxisome**

### Phylogenetic analysis of *APX* genes

**Figure 1:**
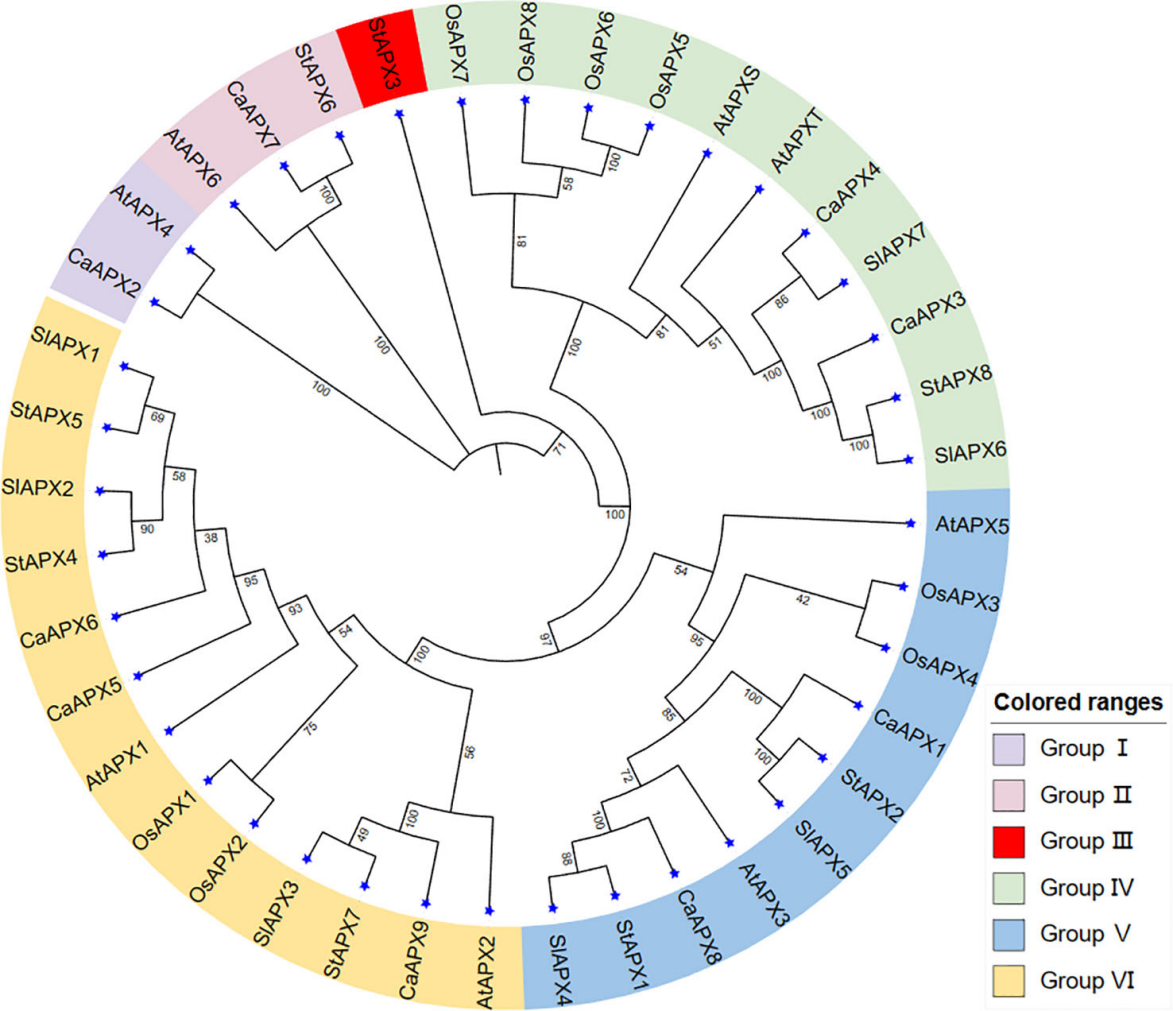
Phylogenetic relationships of *APX* gene family members in five species, including rice, tomato, potato, pepper, and *Arabidopsis thaliana*.

The 40 genes from five species (*Arabidopsis thaliana*, rice, tomato, potato, and pepper) were clustered into six groups (I–VI) (**Figure 1**). The nine *CaAPX* belonged to five groups in the phylogenetic tree, with the exception of group III. Group III only has one gene, *StAPX3*, including the lowest genes among the six groups. Group VI consisted of 13 genes, including the most genes among the six groups, which contained *AtAPX1* and *AtAPX2*; *OsAPX1* and *OsAPX2*; *SlAPX1*, *SlAPX2*, and *SlAPX3*; *StAPX4*, *StAPX5*, and *StAPX7*; and *CaAPX5*, *CaAPX6*, and *CaAPX9*. Groups IV, V, and VI exhibited five species of *APX* genes, while group I had *CaAPX* and *AtAPX*, group II had *CaAPX*, *StAPX*, and *AtAPX*, and group III only had *StAPX*.

This study also found nine pairs of orthologous genes between the various species: *CaAPX2* and *AtAPX4*; *CaAPX7* and *StAPX6*; *StAPX8* and *SlAPX6*; *CaAPX4* and *SlAPX7*; *StAPX1* and *SlAPX4*; *StAPX2* and *SlAPX4*; *StAPX7* and *SlAPX3*; *StAPX4* and *SlAPX2*; and *StAPX5* and *SlAPX1*, respectively. There are six pairs of the nine orthologous genes between the tomato and potato, and it may be noticed that *StAPXs* have a stronger phylogenetic link with the *SlAPXs*. There were also three pairs of paralogous genes discovered only in rice.

### Gene structures and motif analysis of *CaAPX* genes

**Figure 2:**
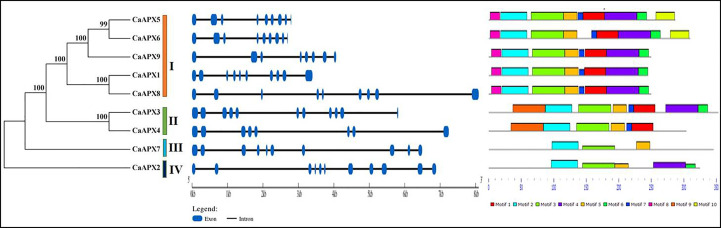
Phylogenetic analysis, gene structures, and conservation motifs of *APX* genes in pepper.

The gene structure of the *CaAPX* genes was guided to obtain insight into the evolution of the *APX* family genes. Moreover, the phylogenetic relationships of the APX proteins in pepper were identified using MEGA 7.0 software. It was to see whether the exon–intron distribution pattern and the phylogenetic tree were in compliance with each other. The results showed that the number of exons ranged from eight to 11, and the number of introns varied from seven to 10, as detailed in **Figure 2**. Group I includes seven to nine introns. Groups III and IV only have one gene, including nine and 10 introns, respectively. While *CaAPX3* and *CaAPX4* showed different exon–intron structures in group II.

The presence of conserved motifs was also identified in nine *CaAPX* genes using MEME. The conserved motif of the *CaAPX* genes explored 64 motifs that varied from three to nine. The motif distributed in the same group is highly similar. Group I includes eight to nine, and group II contains six to eight (**Figure 2**). Motifs 2, 3, and 5 were present in all CaAPX proteins, indicating that these motifs were conserved motifs and functional domains of the APX protein family in pepper. Motif 8 was only discovered in group I, and motif 9 was only present in group II. Overall, as shown in **Figure 2**, the closely related genes of *CaAPX* shared a similar pattern of motif distribution.

### Chromosomal distributions of the *CaAPX* genes

**Figure 3:**
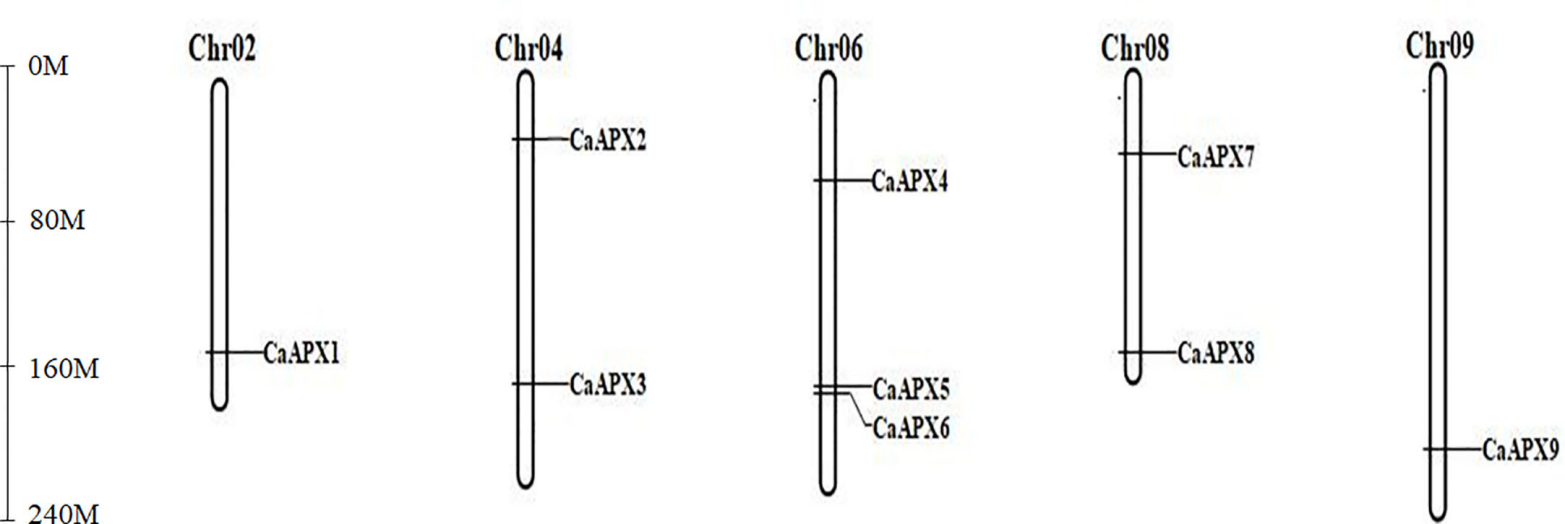
Chromosomal localization of *CaAPX* genes. Nine *CaAPX* genes were distributed unevenly on five chromosomes (Chr02, Chr04, Chr06, Chr08, and Chr09).

The results showed that the *CaAPX* genes were distributed unevenly on five chromosomes, including chromosomes 2, 4, 6, 8, and 9. The other chromosomes did not carry the *APX* genes (**Figure 3**). A maximum of three genes (*CaAPX4*–*CaAPX6*) were localized on chromosome 6, which was followed by chromosome 4 (*CaAPX2* and *CaAPX3*) and chromosome 8 (*CaAPX7* and *CaAPX8*) with two genes. The remaining chromosomes (chromosomes 2 and 9) only consisted of one gene.

### *Cis*-element in the promoters of *CaAPX* genes

**Figure 4:**
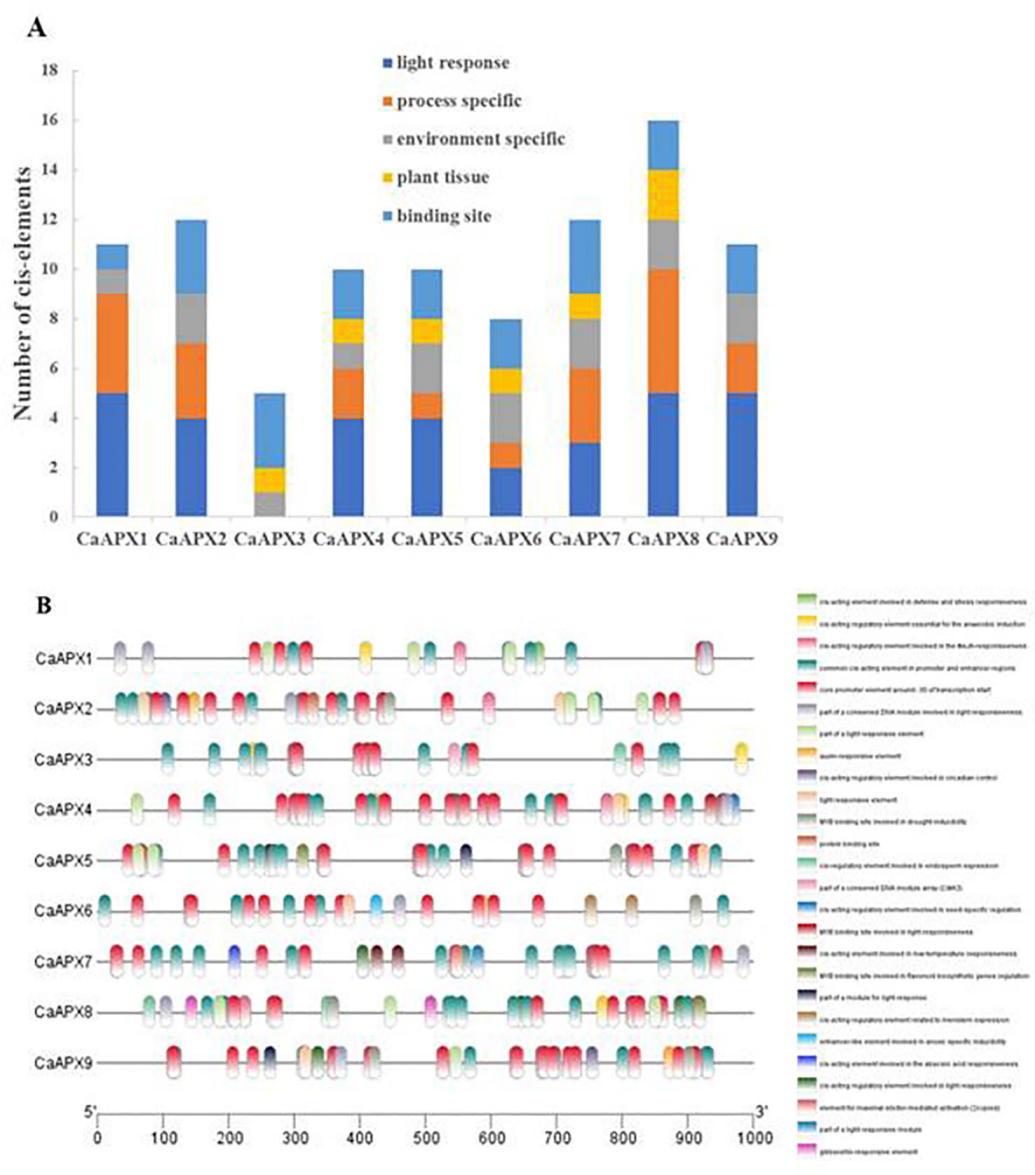
Predicted *cis*-elements in the promoter regions of *CaAPX* genes. **(A)** Number of different *cis*-elements. **(B)** The elements identified are in different colored boxes.

To gain the gene functions and regulations of the *CaAPX* genes, *cis*-elements in *CaAPX* promoter regions were analyzed by searching a 1,000-bp upstream region from each individual *CaAPX* gene’s transcriptional activation site against the PlantCARE database. The results depicted that five major classes of *cis*-elements were present in the promoter region of all *APX* genes: light response; process-specific; environment-specific; plant tissue, and binding site, as shown in **Figure 4**. Among all of the genes except *CaAPX3*, 15 light response elements were found. The results that four phytohormone-correlated (ABA, MeJA, GA, auxin) responsive elements comprising CGTCA-motif, TGACG-motif, p-box, ABRE, and TGA-element were documented. The *cis*-acting regulatory elements involved in environment-specific such as drought (MBS), anoxia (ARE, GC-motif), and low-temperature responsive element (LTR) among all of the genes were found in the promoter region of the *APX* genes of pepper. In addition, four tissue-specific *cis*-elements and five binding site *cis*-elements were also identified.

### Expression profiles of *CaAPX* genes in different tissues

**Figure 5:**
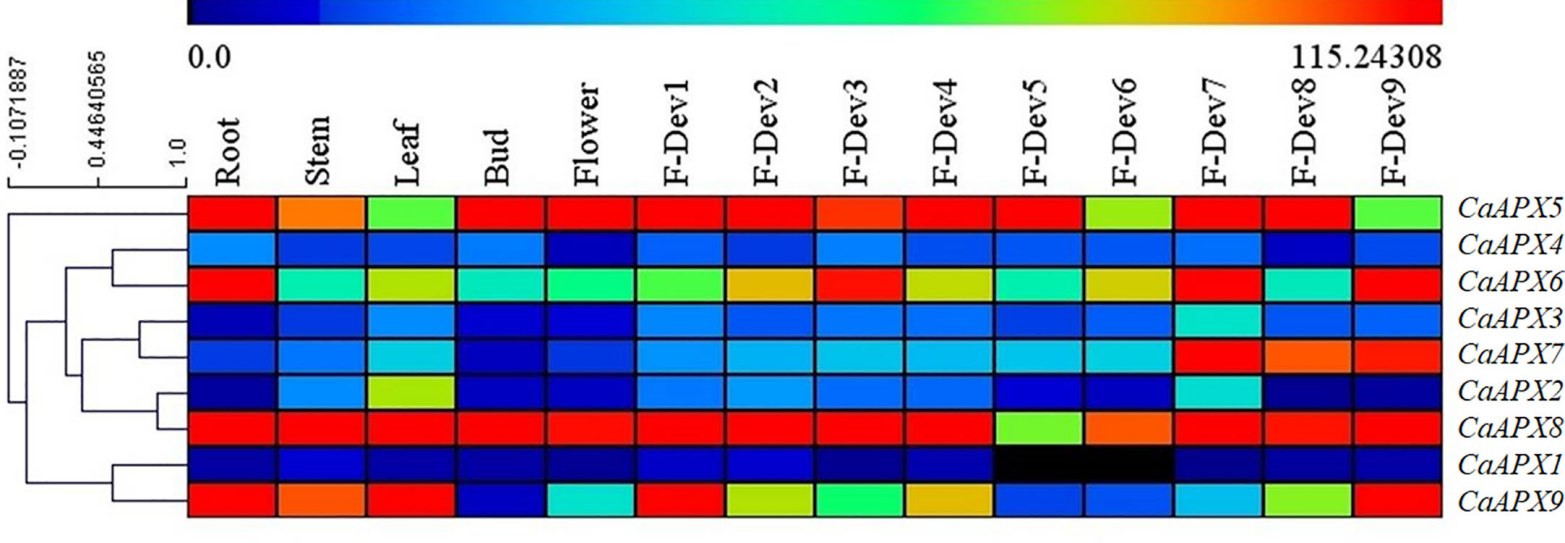
Expression profiles of *CaAPX* genes on RNA-seq in different pepper tissues. The one to nine stages of fruit development are presented in five early stages of color breaking (0–1-, 1–3-, 3–4-, and 4–5-cm-long fruit, and mature-green fruit), the color breaking stage (fruit began to turn red), and three late stages of color breaking (3, 5, and 7 days after color breaking), respectively.

The tissue-specific expression profiles of *CaAPX* genes were investigated at various developmental stages of the pepper cultivar Zunla, as illustrated in **Figure 5**. Results of the study revealed that *CaAPX8* exhibits higher expression levels among all tissues except F-Dev5, implicating the vital roles for pepper, whereas four genes (*CaAPX1*, *CaAPX2*, *CaAPX3*, and *CaAPX4*) showed lower expression in all of the tissues. *CaAPX7* expressed higher values in ripe fruit (F-Dev7, F-Dev8, and F-Dev9), suggesting its participation in fruit development. Among those genes, it was observed that *CaAPX5* had the highest expression levels during the bud stage. The rest of the genes (*CaAPX6* and *CaAPX9*) with the higher expression level displayed tissue specificity in the root and F-Dev9.

### Expression pattern of *CaAPX* genes in response to different abiotic stresses

**Figure 6:**
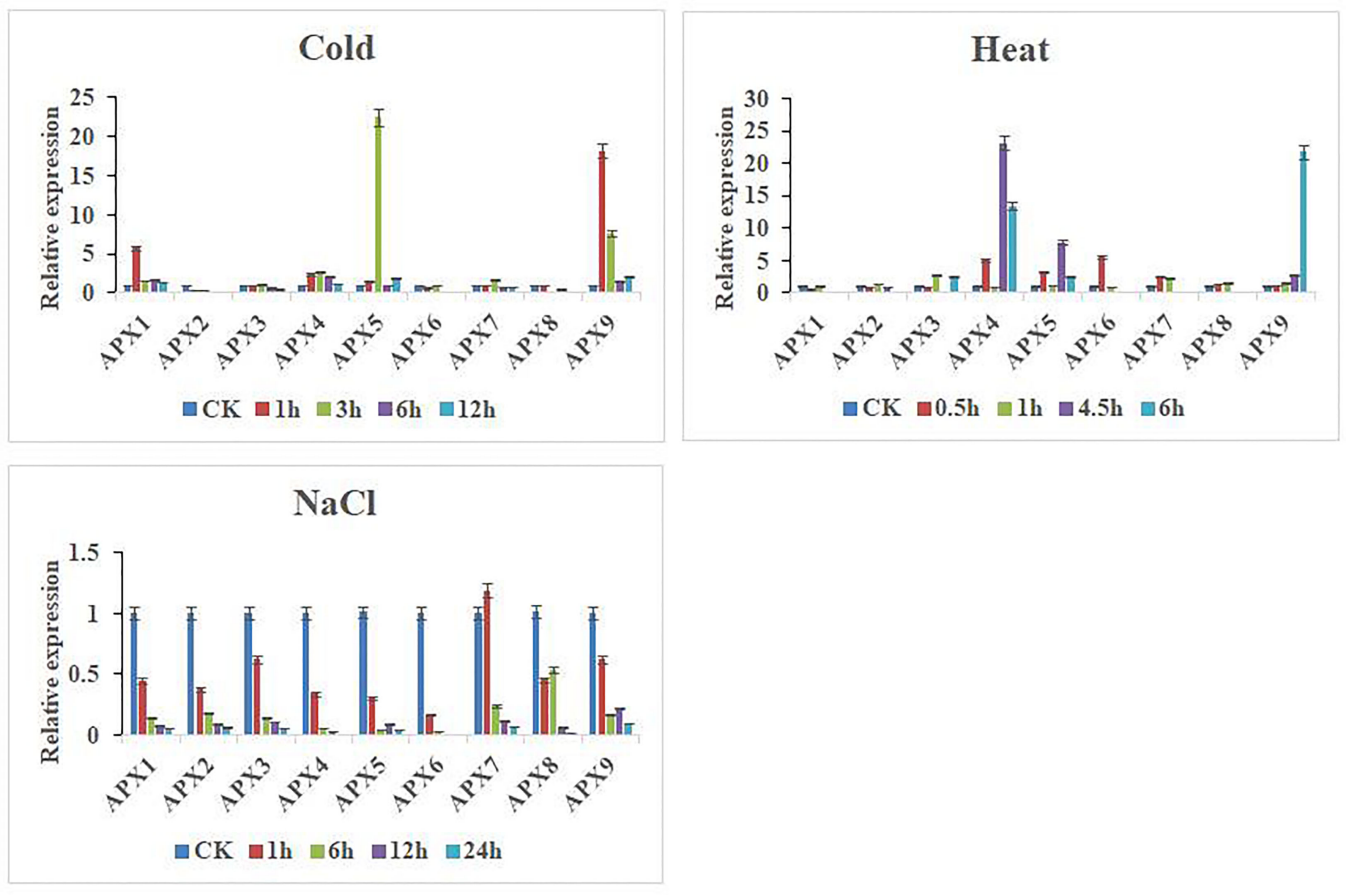
Expression of *CaAPX* genes under cold, heat, and salt stress.

In this study, to further investigate the expression patterns of nine *CaAPX* genes differentially expressed under abiotic stresses, we performed qRT-PCR analysis on the cultivated variety, D50, under cold (4°C), heat (42°C), and NaCl (300 mM) treatment conditions (**Figure 6**). Under cold treatment, *CaAPX1*, *CaAPX4*, *CaAPX5*, and *CaAPX9* were upregulated in response to cold stress at 1, 3, 6, and 12 h as compared to CK, while *CaAPX2* showed continuous downregulation at all treatment points. *CaAPX1* was upregulated (5.81-fold) at 1 h, *CaAPX5* was upregulated (22.45-fold) at 3 h, and *CaAPX9* was upregulated (18.13- and 7.62-fold) at 1 and 3 h, respectively, compared to CK. Under heat treatment, *CaAPX9* showed a continuous upregulation response as compared to CK and showed maximum expression (21.80-fold) at 6 h, while *CaAPX1* was downregulated at all treatment points. *CaAPX4* was upregulated (5.04-, 23.13-, and 13.43-fold) at 0.5, 4.5, and 6 h, *CaAPX5* was upregulated (3.24-, 7.81-, and 2.38-fold) at 0.5, 4.5, and 6 h, and *CaAPX6* was upregulated (5.58-fold) at 0.5 h, respectively, compared to CK. Under NaCl treatment, most of the genes were downregulated, especially *CaAPX1*, *CaAPX2*, *CaAPX3*, *CaAPX4*, and *CaAPX6*, which were continuously downregulated at all treatment points, except *CaAPX7*, which showed a slightly higher expression at 1 h.

## Discussion

APX is one of the key enzymes for active oxygen removal in plants, playing an important role in plant growth and development as well as stress response. With the development of sequenced genomes, *APX* gene families have been found in many plants, such as *Arabidopsis* (Panchuk et al., 2002), rice (Teixeira et al., 2004), upland cotton (Tao et al., 2018), tomato (Najami et al., 2008), and so on. According to the subcellular localization, APX can be divided into cytoplasmic APX, peroxisome APX, chloroplast APX, and mitochondrial APX, but the localization and quantity of APXs in cells are different in different species. Previous studies have shown that different types of *APX* genes have different functions. For example, chloroplast APX is mainly used to protect the photosynthetic system, while mitochondrial APX plays a positive role in removing hydrogen peroxide generated by fatty acid β oxidation (Renu et al., 2011; Andreia et al., 2014), the cytoplasmic *OsAPX2* gene plays an important role in maintaining H_2_O_2_ homeostasis (Wu et al., 2018), silened peroxisome *OsAPX4* can lead to premature aging of rice (Guan et al., 2010). In this work, nine *APX* genes were found in the pepper genome and divided into four subfamilies. The results of the phylogenetic tree showed that five genes of subfamily I were located in the peroxisome, two genes of subfamily II were located in chloroplasts/mitochondria, one gene of subfamily III was located in cytoplasm and extracell, and one member of subfamily IV was only located in chloroplasts, indicating that the close relatives may have had the same subcellular localization results (**Figure 2**). *CaAPX3* and *CaAPX4* of subfamily II were all located in two chloroplast/mitochondrial organelles, as well as in previous studies of *PtrAPX1* and *PtrAPX5* in *Populus trichocarpa* (Leng et al., 2021). *CaAPX* genes located in peroxisome all contain eight different conserved motifs and have eight to ten exon regions. Rice is a monocotyledonous plant, while *Arabidopsis thaliana*, tomato, potato, and pepper belong to the dicotyledonous family. Therefore, the phylogenetic tree of *Arabidopsis thaliana*, rice, tomato, potato, and pepper was constructed, and it was found that the *APX* gene of pepper is further related to the homologous evolution of rice.

The promoter *cis*-acting element plays an important role in regulating plant biological processes, such as participating in exogenous hormone induction and abiotic stress response (Yang et al., 2019). In this study, it was found that *CaAPX* genes contain hormone-induced related and stress regulatory elements, such as CGTCA-motif, TGACG-motif, TGA-element, ABRE, CGTCA-motif, TCA-element, ARE, MBS, and LTR, through the 1,000-bp upstream ATG sequence analysis of the *CaAPX* family members. Every *CaAPX* gene contains at least one process-specific and one environment-specific class, except *CaAPX3*, which has no hormone-induced *cis*-element, suggesting that *CaAPXs* play an important role in plant hormone and abiotic stress responses.

More and more evidence suggested that *APX* genes may exhibit different expression profiles in different organs/tissues and stress environments. Therefore, the tissue-specific expression of *CaAPX* genes was displayed in eight diverse developmental tissues using RNA-seq data (**Figure 5**), and these results are consistent with previous reports. For example, *AtAPX1* is involved in many biological processes (Pnueli et al., 2003; Davletova et al., 2005) and is constitutionally higher expressed in plant roots, stems, leaves, and many other tissues (Zimmermann et al., 2004). The expression of *AtAPX6* was very high at the seed postmaturity stage (Chen et al., 2014). Maize *APXb1* is highly expressed in roots, shoots, and immature leaves, and *APXb2* is abundantly expressed in leaves and reproductive organs (Qu et al., 2020). In our study, *CaAPX8* and *CaAPX5* presented higher expressions in most tissue, suggesting two genes involved in many biological processes. The expression levels of *CaAPX1*, *CaAPX2*, *CaAPX3*, and *CaAPX4* exhibited very low levels during the most normal growth and development of pepper. These results indicate that the four genes may play a small role in the normal growth and development of plants. *CaAPX7* expression in pepper increased significantly with fruit maturation. These results suggest that *APX7* may play a key role in fruit development.

In this study, qRT-PCR was used to appraise the expression patterns of *CaAPXs* under low temperature, high temperature, and salt stress (**Figure 6**). Under the three abiotic stresses, the expression patterns of *CaAPXs* were different, *CaAPX5* and *CaAPX9* showed upregulated expression under low temperature, and *CaAPX4* and *CaAPX9* showed higher expression under high temperature, indicating that *CaAPXs* may play an important role in the abiotic stress response of pepper, which was consistent with previous research results (Sharma and Dubey, 2005; Koussevitzky et al., 2008). However, the expression levels of some genes showed a downregulated expression under stress. For example, under salt stress, the expression levels of all the *CaAPXs* except *CaAPX7* at 1 h were downregulated at all times. It is speculated that the expression of these genes was inhibited after stress and that ROS is cleared by other genes or by other means. In addition, the expression patterns of individual genes were different under different stresses. The expression level of *CaAPX1* was significantly upregulated in low-temperature stress but downregulated in high-temperature stress, suggesting that *CaAPX* genes played different functions under different stresses. The result agreed with earlier discoveries; for example, *cAPX* expression of *Spinacia oleracea* was upregulated under strong light and ultraviolet light, but the transcription level did not change under drought and salt stress. The transcription levels of *mAPX*, *sAPX*, and *tAPX* did not change after ABA, drought, strong light, and salt treatment (Yoshimura et al., 2000). The results of qRT-PCR showed that *APX* genes were induced to express under high temperatures, and *CaAPX5* and *CaAPX9* showed significantly upregulated expression every time. By constructing a phylogenetic tree, we found that *CaAPX5*, *CaAPX9*, and *AtAPX2* are clustered into a cluster that is close in evolutionary relationship. Studies have shown that *AtAPX2* plays a very important regulatory role in the process of heat stress response at different developmental stages of plants (Shi et al., 2001; Frank et al., 2009). These discoveries offered compelling evidence that *APX* genes play a well-maintained role in defending diverse plant species against abiotic stresses. In the future, we could validate the function of *CaAPX* genes using overexpression and the CRISPR/Cas system and provide a basis study for breeding pepper.

## Data availability statement

The original contributions presented in the study are included in the article/**Supplementary Material**. Further inquiries can be directed to the corresponding authors.

## Author contributions

Conceived and designed the experiments: XP and HW. Performed the experiments: JC, YX, JL, and YZ. Analyzed the data: XP, YX, LW, and JZ. Wrote the paper: XP, YX, and HW. All authors contributed to the article and approved the submitted version.
