# Supplementary material for: Genome-wide characterization of ascorbate peroxidase gene family in pepper (Capsicum annuum L.) in response to multiple abiotic stresses
Source: Front Plant Sci. 2023 May 8;14:1189020. doi: 10.3389/fpls.2023.1189020 (PMC10210635; doi:10.3389/fpls.2023.1189020)
Supplement: Supplementary file 1 [file Table_1.doc]

Supplemental Table S1 Primer information of CaAPX genes in pepper.

>CaAPX01

F1：ACAATTCCGCCGTTATGTGG

R1：AACAGCAACTCCAACGACAC

>CaAPX2

F2：CCCTCGTCAGCTAGCAGTAA

R2：GTAGCAAGTAGGGCCTCTGT

>CaAPX3

F3：ATGAGAGCATCGAGGACTGG

R3：CTCCAGCCTCCTCAATAGCA

>CaAPX4

F4：CAGTCGTTTGCTCAGTTCGT

R4：GTCATTGTCGTAGGCGTGAC

>CaAPX5

F5：AGCCCATTAGGGAGCAGTTT

R5：GGTTCTGGCTTGTCCTCTCT

>CaAPX6

F6：AAACTGAGCAGTCTCACGGA

R6：GTCCTCCAGTCACCTCAACA

>CaAPX7

F7：TGGTCCAATTCGAGCTTCCT

R7：ACGGCACAAGAGACAAGGTA

>CaAPX8

F8：GGTGTTGTTGCTGTTGAGGT

R8：TGTGGCACACCTTGTTTAGC

>CaAPX9

F9：TCTTGACATTGCCGTTCGAC

R9：GTGTCTTGTCCTGTCTCCCA
